# Comprehensive Analysis of LincRNAs in Classical and Basal-Like Subtypes of Pancreatic Cancer

**DOI:** 10.3390/cancers12082077

**Published:** 2020-07-27

**Authors:** Markus Glaß, Agnes Dorn, Stefan Hüttelmaier, Monika Haemmerle, Tony Gutschner

**Affiliations:** 1 Institute of Molecular Medicine, Section for Cell Biology, Medical Faculty, Martin-Luther University Halle-Wittenberg, 06120 Halle/Saale, Germany; markus.glass@medizin.uni-halle.de (M.G.); stefan.huettelmaier@medizin.uni-halle.de (S.H.); 2 Institute of Pathology, Section for Experimental Pathology, Medical Faculty, Martin-Luther University Halle-Wittenberg, 06120 Halle/Saale, Germany; agnes.dorn@uk-halle.de; 3 Junior Research Group ‘RNA Biology and Pathogenesis’, Medical Faculty, Martin-Luther University Halle-Wittenberg, 06120 Halle/Saale, Germany

**Keywords:** cancer, lincRNA, lncRNA, miRNA, pancreatic ductal adenocarcinoma, RBP

## Abstract

Pancreatic ductal adenocarcinomas (PDAC) belong to the deadliest malignancies in the western world. Mutations in TP53 and KRAS genes along with some other frequent polymorphisms occur almost universally and are major drivers of tumour initiation. However, these mutations cannot explain the heterogeneity in therapeutic responses and differences in overall survival observed in PDAC patients. Thus, recent classifications of PDAC tumour samples have leveraged transcriptome-wide gene expression data to account for epigenetic, transcriptional and post-transcriptional mechanisms that may contribute to this deadly disease. Intriguingly, long intervening RNAs (lincRNAs) are a special class of long non-coding RNAs (lncRNAs) that can control gene expression programs on multiple levels thereby contributing to cancer progression. However, their subtype-specific expression and function as well as molecular interactions in PDAC are not fully understood yet. In this study, we systematically investigated the expression of lincRNAs in pancreatic cancer and its molecular subtypes using publicly available data from large-scale studies. We identified 27 deregulated lincRNAs that showed a significant different expression pattern in PDAC subtypes suggesting context-dependent roles. We further analyzed these lincRNAs regarding their common expression patterns. Moreover, we inferred clues on their functions based on correlation analyses and predicted interactions with RNA-binding proteins, microRNAs, and mRNAs. In summary, we identified several PDAC-associated lincRNAs of prognostic relevance and potential context-dependent functions and molecular interactions. Hence, our study provides a valuable resource for future investigations to decipher the role of lincRNAs in pancreatic cancer.

## 1. Introduction

Pancreatic cancer with its most common type, pancreatic ductal adenocarcinomas (PDAC), is currently the fourth leading cause of cancer-related deaths in developed countries [1]. Dismal prognosis of disease has several reasons. First, pancreatic cancer is typically diagnosed in late stages due to non-specific symptoms or a lack thereof as well as the unavailability of sensitive and specific biomarkers in conjunction with difficulties in imaging early stage tumours. Second, PDAC is a highly aggressive disease with extensive local growth and early distant metastases, precluding many patients from surgery. Third, pancreatic cancer is characterized by a high degree of resistance to currently available treatment options, i.e., chemotherapy, radiotherapy, or targeted therapies. All these factors result in a 5-year survival rate below 5%, if surgical resection is not possible [2]. If prognosis will not improve, pancreatic cancer is predicted to be the second leading cause of death in the next decade [1]. Molecularly, most PDACs are associated with somatic mutations, particularly affecting the KRAS, TP53, CDKN2A, and SMAD4 genes. Especially, KRAS mutations occur in more than 90% of all tumour samples [3,4]. In transgenic mouse models, activating mutations in the Kras gene were shown to be sufficient to induce PDAC [5]. Even in the few KRAS wild-type tumours, somatic mutations activating the RAS-MAPK pathway up- or downstream of KRAS have been identified [3]. However, these known and frequent mutations did not lead to clinically valuable tumour classifications accounting for different patient survival and therapy response, suggesting that origins of PDAC heterogeneity may be found at the post-genetic level [6]. Multiple transcriptomic studies provided valuable insights into pancreatic carcinogenesis and identified disease subtypes of prognostic relevance. At least three subtype classifications based on transcriptomic data have been proposed. Moffit et al. [7] described two subtypes obtained via non-negative matrix factorization (NMF) of microarray data; a basal-like subtype, generally associated with worse outcome and a classical subtype. Furthermore, they derived two classes of stroma subtypes, normal and activated, yielding four classes for distinguishing primary tumour samples. Collisson et al. [8] suggested three subtypes also generated by NMF of microarray data: classical, quasi-mesenchymal and exocrine-like. Notably, the gene signature derived from the classical subtype showed high overlap with the signature derived from the classical subtype of Moffitt et al. [7]. Moreover, Bailey et al. [9] identified four subclasses by applying NMF to RNA-seq and microarray data of pancreatic cancer samples: squamous, pancreatic progenitor, immunogenic and aberrantly differentiated endocrine exocrine (ADEX). At least part of the differences of classifications are a consequence of different input material, either using bulk tumour tissue containing cells of the tumour microenvironment [7,9] or microdissected tumour epithelium [8] resulting in differences in tumour purity. In line with this, The Cancer Genome Atlas (TCGA) research network applied all three classification systems to their own RNA expression data and concluded that high purity tumours can be consistently classified into a basal-like/squamous group and a classical/progenitor group. In contrast, the strong association of immunogenic or ADEX as well as exocrine-like or quasimesenchymal subtypes with low purity samples within the TCGA cohort suggested that these subtypes may reflect gene expression from non-neoplastic cells [3]. Furthermore, the TCGA analysis found that the basal-like and classical subtype are characterized by expression differences of non-coding RNAs (ncRNAs), including microRNAs (miRNAs) and long non-coding RNAs (lncRNAs). LncRNAs can function as regulators of gene expression acting on the epigenetic, transcriptional as well as on the post-transcriptional level [10,11]. Several lncRNAs have been implicated in pancreatic cancer acting, e.g., as competitive endogenous RNAs (ceRNAs) that interfere with miRNA-dependent regulation [12,13,14,15] or as part of lncRNA signatures predicting PDAC patient survival [16,17,18,19]. However, an in-depth characterization of the lncRNA landscape of different PDAC subtypes, including a description of their potential interactions as well as their correlation with other protein-coding and non-coding genes, has not been systematically performed yet. Here, we aim to provide a comprehensive overview of a class of lncRNAs called long intervening RNAs (lincRNAs), which are lncRNAs that do not overlap exons of either protein-coding or other non-lincRNA types of genes [20]. LincRNAs are often transcribed by RNA Polymerase II and thus are usually capped and polyadenylated allowing their detection in large-scale RNA-sequencing-based cancer transcriptome data sets commonly generated using a polyA-selection step. Importantly, due to their non-overlapping genomic localization lincRNA expression patterns are easier to interpret than those of transcripts from loci overlapping other gene classes. Furthermore, they are amenable to specific genetic perturbations using genome engineering tools to study their cellular and organismal functions [21,22]. Hence, our analysis highlights disease-associated lincRNAs as well as their putative molecular interactions and thereby provides a valuable resource and an ideal starting point for future investigations to expand our knowledge about the onset and progression of pancreatic cancer.

## 2. Results

### 2.1. LincRNA Expression Is Altered in PDAC Compared to Normal Pancreas

**Figure 1:**
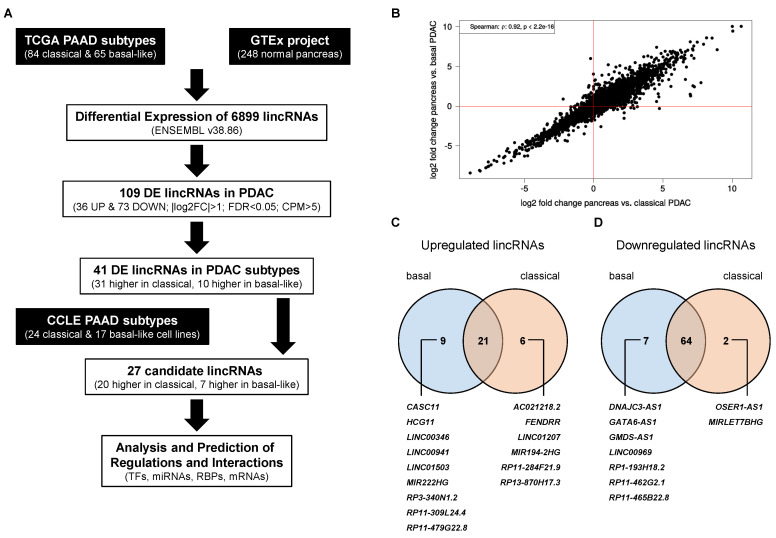
Analysis of lincRNA expression in PDAC (pancreatic ductal adenocarcinomas). (**A**) Flow-chart outlining the analysis pipeline to identify differentially expressed lincRNAs in pancreatic cancer using TCGA (The Cancer Genome Atlas) tumour samples; (**B**) $log_{2}$ fold changes of 6899 annotated lincRNAs in the comparison between normal pancreas tissue and classical PDAC (x-axis) and between pancreas tissue and PDAC samples categorized as basal-like subtype (y-axis). (**C**) Venn diagram depicting the overlap of lincRNAs upregulated in basal-like and classical tumour subtypes, respectively. (**D**) Venn diagram showing the overlap of lincRNAs downregulated in basal-like and classical PDAC subtypes. In total, 109 lincRNAs fulfilled the selection criteria ($|log_{2}FC|>1$; False Discovery Rate (FDR) <0.05; avg. Counts per million (CPM)>5 in normal pancreas or the respective tumour subtype). PAAD: Pancreatic adenocarcinoma; GTEx: Genotype-Tissue Expression; CCLE: Cancer Cell Line Encyclopedia; TFs: Transcription factors; RBPs: RNA-binding proteins.

To gain insights into the expression landscape of lincRNAs in pancreatic cancer, we obtained RNA sequencing data from The Cancer Genome Atlas Pancreatic adenocarcinoma (TCGA PAAD) project [3]. However, since these data contained only four normal tissue samples, we compared the primary tumour samples with pancreas RNA-seq data from the Genotype-Tissue Expression (GTEx) project [23]. We used raw count data from both projects and processed them together to avoid RNA composition biases (see Methods). Altogether, we compared 248 normal pancreas samples against 84 classical subtype and 65 basal-like subtype tumour samples (Figure 1A). Gene set enrichment analyses (GSEA, [24]) using the fold changes obtained from the differential expression analyses showed pancreatic cancer related gene sets among the most significantly enriched sets supporting the cogency of this comparison (Figure S1A).

**Figure 2:**
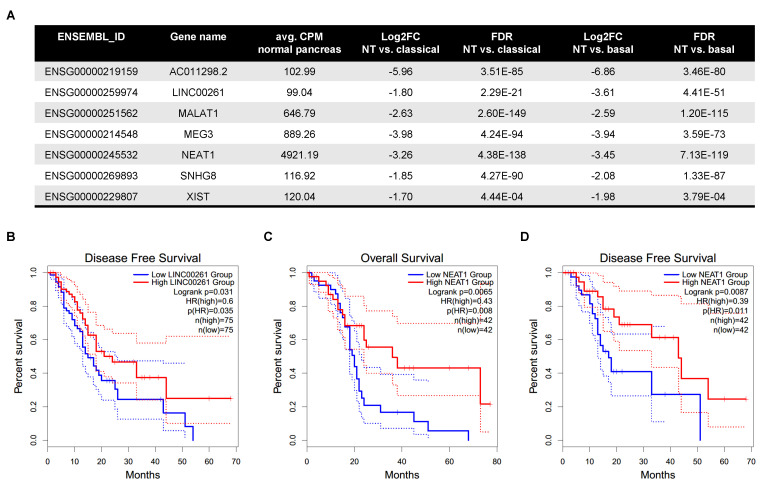
Expression and prognostic relevance of highly abundant lincRNAs. (**A**) Table shows the expression and regulation ($|log_{2}FC|>1$; FDR <0.05) of highly expressed lincRNAs (avg. CPM >99 in normal pancreas). (**B**,**D**) Overall and disease-free survival analysis (OS and DFS, respectively) based on lincRNA expression was performed on either both, basal and classical TCGA PAAD samples together or separately using GEPIA2 ([36]). Log-rank test (Mantel-Cox) was used for hypothesis testing. The cox proportional hazard ratio (HR), logrank *p*-value and the 95% confidence interval information is included in the graphs. (**B**) In general, low expression of LINC00261 is associated with worse DFS of PDAC patients. (**C**,**D**) Low expression of NEAT1 is specifically associated with a poor OS (**C**) as well as DFS (**D**) in the classical subtype.

Next, we gathered expression data of 6899 individual lincRNA genes (Table S1) and compared their respective tumour subtype-specific expression to normal pancreatic tissue. In general, the $log_{2}$ fold changes observed in both expression analyses showed a strong and highly significant correlation Spearman ρ=0.92, $FDR<2\times 10^{-16}$; Figure 1B) indicating similar expression trends of lincRNAs in both subtypes. However, application of filters for fold change ($|log_{2}FC|>1$), significance (False Discovery Rate (FDR) <0.05) and absolute expression level (average counts per million (CPM) ≥5 in either pancreas or the respective tumour samples of each subtype) revealed subtype-specific differences and identified a total of 109 differentially expressed lincRNAs (Figure S1B,C). Of note, the number of downregulated lincRNAs was more than twice the number of upregulated lincRNAs in both comparisons. Specifically, in tumours assigned to the classical subtype 93 lincRNAs showed a significantly altered expression level with 27 being up- and 66 being downregulated. In the basal-subtype group 101 lincRNAs were changed and 30 lincRNAs had a higher whereas 71 lincRNAs had a lower expression in the tumour tissue. Comparison of both lists of differentially expressed lincRNAs identified commonly deregulated transcripts as well as some candidates altered in a subtype-specific manner (Figure 1C,D). However, a closer examination of the latter cases revealed similar expression trends in the large majority of these lincRNAs yet their absolute expression level or their fold change prevented their inclusion in the list of differentially expressed lincRNAs in one subtype, but not the other. For example, FENDRR is highlighted as a lincRNA that seemed to be specifically upregulated in the classical subtype (Figure 1C). However, its expression was also significantly increased in the basal-like subtype ($log_{2}FC=1.49$, $FDR=5.56\times 10^{-23}$), but due to its low average expression level, which exceeded 5 CPM only in the classical subtype, FENDRR was not included in the list of deregulated lincRNAs in basal-like tumours besides showing the same overall expression trend. Nevertheless, further analysis identified three lincRNAs (CASC11, RP11-465B22.8, LINC01207) out of the initial 109 which showed a significant expression change in one subtype compared to normal pancreas that was not observed in the other subtype. In detail, CASC11 was strongly upregulated in the basal-like subtype ($log_{2}FC=6.01$; $FDR=2.60\times 10^{-129}$), but slightly, yet not significantly downregulated in the classical subtype ($log_{2}FC=-0.2$; FDR=0.54) whereas RP11-465B22.8 showed a decreased expression ($log_{2}FC=-1.14$; $FDR=1.84\times 10^{-14}$) specific to the basal-like subtype only. In contrast, the upregulation of LINC01207 ($log_{2}FC=2.24$; $FDR=6.35\times 10^{-33}$) was specific to the classical subtype whereas its expression virtually did not change in the basal-like subtype. These specific expression patterns suggest that these lincRNAs might have context-dependent functions. In contrast, our analysis identified 85 lincRNAs that were commonly deregulated in both subtypes (21 up-/64 downregulated) suggesting a rather general role for the biology of PDAC. Hence, these lincRNAs would be interesting candidates to follow-up on. In order to further narrow down the list of 85 lincRNAs, we applied a more stringent filter to retrieve candidates with a robust expression level (average CPM > 99). This left us with seven differentially regulated lincRNAs, which are all commonly downregulated in tumours compared to normal pancreas (Figure 2A). The strongest decrease was observed for AC011298.2 ($log_{2}FC=-5.96$/−6.86), a lincRNA of unknown function that was previously included in a six-lncRNA signature to predict survival of patients with bladder urothelial carcinoma [25]. Moreover, we observed a decrease in the expression of SNHG8, a host gene for a small nucleolar RNA (snoRNA), which was recently shown to be increased in PDAC, associated with an adverse prognosis [26]. In addition, we confirmed the reduced expression of LINC00261 and MEG3, which are thought to function as tumour-suppressive lincRNAs in pancreatic cancer, and especially in neuroendocrine neoplasms in the case of MEG3 [27,28,29,30,31]. We also detected a strong reduction of XIST, NEAT1 and MALAT1, three conserved nuclear lncRNAs with well-known links to a broad range of cancer types [32,33,34,35]. To further evaluate the clinical and prognostic relevance of these seven highly expressed and deregulated lincRNAs we performed survival analyses using the GEPIA2 data portal (gepia2.cancer-pku.cn [36]). This analysis revealed striking associations between the expression of LINC00261 and NEAT1 with overall (OS) and/or disease-free survival (DFS) in TCGA PDAC patient samples. In detail, low expression of LINC00261 was predictive of poor DFS (Figure 2B) across all 150 samples, whereas low levels of NEAT1 were associated with worse OS (Figure 2C) and DFS (Figure 2D) in classical subtype samples. Hence, based on their abundance, significant downregulation and their prognostic relevance, LINC00261 and NEAT1 might be interesting candidates for functional follow-up studies to dissect their cellular and molecular functions in pancreatic cancer.

### 2.2. LincRNAs Are Differentially Expressed between PDAC Subtypes

**Figure 3:**
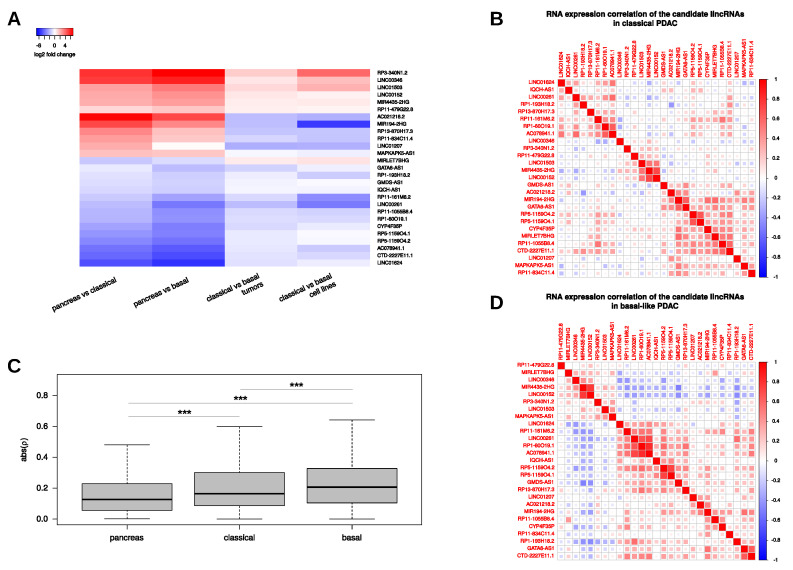
Expression of 27 differentially expressed and subtype-related candidate lincRNAs. (**A**) Heatmap displays log2 fold changes of 27 lincRNAs that show expression differences between tumour subtypes and normal pancreas as well as between both cancer subtypes in situ and in cellulo. (**B**,**D**) Spearman correlation coefficients (ρ) of the selected lincRNAs in classical (**B**) and basal-like (**D**) PDAC samples. (**C**) Distribution of magnitudes of Spearman correlation coefficients (|ρ|) obtained from correlation tests between the selected lincRNAs in pancreas and PDAC subtype samples.

As discussed above, our subtype-resolved gene expression analysis identified commonly deregulated lincRNAs as well as subtype-specific expression differences that might contribute to the intrinsic molecular and prognostic differences between the two subtypes. We analyzed the differential expression of lincRNAs in more detail in order to identify candidate lincRNAs whose function or regulation may depend on or contribute to the respective disease context. To this end, we considered the aforementioned 109 PDAC-associated lincRNAs (see Figure S1C) and compared their expression in the classical subtype with their respective level in the basal-like subtype. This analysis identified a total of 41 lincRNAs significantly differentially expressed (FDR<0.05) between both pancreatic cancer subtypes. Next, we integrated RNA-seq data from the Cancer Cell Line Encyclopedia (CCLE, ref. [37]) and examined which of the 41 lincRNAs found to be deregulated in situ showed the same trend of deregulation in cellulo when comparing PDAC cell lines classified as “classical” or “basal-like” (Table S2) [38]. This analysis resulted in a list of 27 PDAC-associated lincRNAs that were consistently higher/lower expressed in one of the two PDAC subtypes (Figure 3A, Table S3). Of those, 7 lincRNAs were generally higher expressed in cell lines and tissues assigned to the basal-like subtype while the remaining 20 lincRNAs were higher expressed in the classical subtype samples. For example, our analysis identified LINC01207 to be upregulated in PDAC which is in line with an earlier study that reported an increased expression of LINC01207 in pancreatic adenocarcinoma as well as a putative role of this lincRNA in regulating apoptosis and autophagy [39]. However, our disease subtype comparison implicated a stronger biological relevance of LINC01207 in tumours of the classical subtype. On the other side, the highly expressed lincRNA LINC00261 was found to be generally downregulated in both cancer subtypes and this decrease was more pronounced in the basal-like subtype tissues and cell lines. LINC00261 is suggested to act as a tumour-suppressive lincRNA in lung [40], liver [41] and gastric cancer [42]. In pancreatic cancer, LINC00261 was implicated in the epithelial-to-mesenchymal transition (EMT) pathway, which is crucial for metastasis and disease progression [27,43]. Intriguingly, LINC00261 might also be important for human endoderm differentiation [44] and its reduced expression is a predictor of DFS (see Figure 2B). Additional lincRNAs whose expression is associated with OS and DFS either across all TCGA PDAC samples or only in a certain subtype are shown in Figures S2 and S3. For example, another lincRNAs of prognostic relevance that we identified with our analysis pipeline was GATA6-AS1. This lincRNA was significantly lower expressed in the basal-like subtype samples and its low expression in PDAC was associated with worse OS (Figure S2C,F) and DFS (Figure S3G,L) in the complete TCGA PDAC cohort as well as in the basal-like group. Next to LINC00261 and GATA6-AS1, which were both downregulated in the basal-like subtype, our analysis also unveiled lncRNAs with a significant upregulation in the basal-like subtype. For example, RP3-340N1.2, a novel uncharacterized and spliced lincRNA, was strongly increased in both tumour subtypes with a more than 2-fold higher expression in basal-like tumours compared to classical ones. Even more pronounced expression changes were observed for LINC00152, also referred to as CYTOR, whose herein described upregulation in pancreatic cancer confirmed a recent finding using a small cohort of six PDAC and five control tissues [45]. While functional analyses of LINC00152 in the context of pancreatic cancer are currently lacking, this lincRNA was previously shown to contribute to cancer progression acting as an oncogenic lincRNA in diverse tumour types [46]. Our survival analysis revealed that high expression of LINC00152 in PDAC is associated with a poor DFS (Figure S3H,M) suggesting a putative tumour-promoting role of LINC00152 also in pancreatic cancer, especially in the basal-like subtype.

To further characterize the expression and to identify common expression patterns among the 27 selected lincRNAs, we performed correlation analyses (Figure 3B,D). Consideration of the magnitudes of the Spearman correlation coefficients revealed a steady increase from the normal pancreas to the basal-like subtype indicating strongest associations between our candidate lincRNAs in this subtype, regardless of the direction (Figure 3C). The strongest correlation in the classical subtype was found between LINC00152 and MIR4435-2HG (Spearman ρ=0.72; Figure 3B). A similar strong correlation was also found in the basal-like subtype (ρ=0.78) as well as in normal pancreas (ρ=0.76) (Table S4). Intriguingly, both lincRNA genes are located on chromosome 2 and MIR4435-2HG was recently found to be a paralog of LINC00152 differing only by 13 exonic single nucleotide exchanges [47].

Since both lincRNA loci are highly similar, including their up- and downstream genomic sequences, the high correlation seen in all tissue samples might be due to shared transcriptional or similar post-transcriptional mechanisms regulating transcript production and/or decay. Another strong positive correlation seen in all tissues yet most pronounced in normal pancreas (ρ=0.87) existed between RP5-1159O4.1 (AC004982.2) and RP5-1159O4.2 (AC004982.1), which are both located in close proximity on chromosome 7, again indicating a common (transcriptional) regulation. On the other side, the strongest positive correlation in the basal-like subtype was found between AC078941.1 and RP1-60O19.1 (ρ=0.83) that do not share a common genomic locus (Figure 3D). Interestingly, the correlation between these lincRNAs was considerably lower in the classical subtype samples (ρ=0.63) and could not be detected in normal pancreas indicating tumour- and partially subtype-specific regulatory mechanisms responsible for the observed downregulation of both lincRNAs in neoplastic lesions (Table S4). On the opposite side, the strongest negative correlation between two lincRNAs in the basal-like subtype was observed between GMDS-AS1 and MIR4435-2HG (ρ=−0.49). Not surprisingly, a similar strong negative correlation was observed between GMDS-AS1 and LINC00152 (ρ=−0.43). Interestingly, both negative correlations were considerably attenuated in the classical subtype (ρ=−0.24 and −0.12, respectively) and normal pancreas (ρ=−0.12 and −0.19, respectively). Another example for negative correlation with a stronger emphasis in the basal-like subtype was present between LINC00261 and LINC00152 (classical: −0.22, basal: −0.39), lending further support to the idea of a putative opposing (i.e., oncogenic) role of LINC00152 in pancreatic cancer. Of note, MIR4435-2HG showed a very similar negative correlation to LINC00261 and might also function as a tumour-promoting lincRNA, an idea that is supported by its prognostic relevance for DFS in TCGA PDAC samples (Figure S3I). Last but not least, another strong negative correlation existed between RP1-193H18.2 and AC021218.2 (ρ=−0.43) which was highly specific to the classical subtype (normal: ρ=−0.07; basal-like: ρ=−0.03). Of note, high expression of AC021218.2 in classical subtype tumours was associated with worse OS (Figure S2D). While the underlying mechanisms and the disease relevance await further investigations, this example of a strong negative correlation, which would have gone unnoticed in a global PDAC expression analysis, underscores the value of performing subtype-resolved transcriptome analyses to gain a deeper understanding of the biological processes contributing to carcinogenesis.

### 2.3. Putative Transcriptional and Post-Transcriptional Regulators of LincRNA Expression

**Figure 4:**
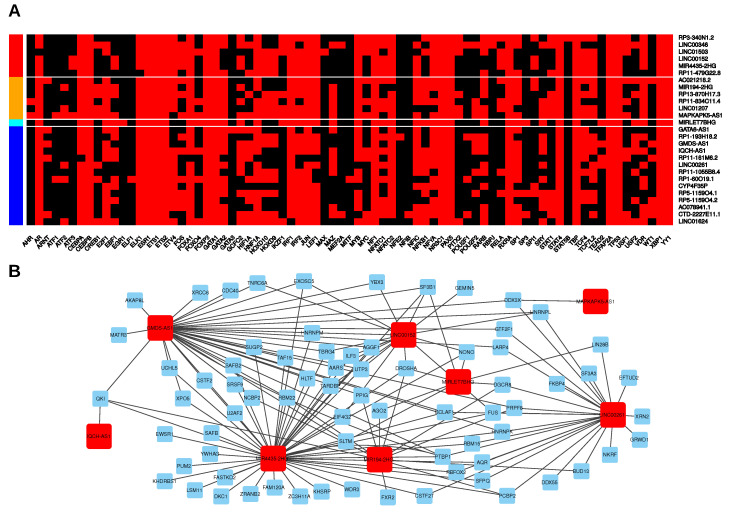
Putative transcriptional and post-transcriptional regulators of lincRNAs. (**A**) Transcription factors (TFs) predicted to bind to the promotor region ranging from 2000 bp upstream to 1000 bp downstream of the putative transcriptional start site (TSS) of the respective lincRNA. Red colour denotes a predicted TF binding. Row colour codes on the left side denote lincRNAs upregulated in both tumour subtypes compared to normal pancreas with significantly higher expression in the basal-like subtype (red); upregulated in both subtypes and significantly lower in the basal-like subtype (orange); downregulated in both subtypes and significantly higher in the basal-like subtype (cyan); downregulated in both subtypes and significantly lower in the basal-like subtype (blue). (**B**) Network representation of potential interactions between RNA-binding proteins (RBPs) and the selected candidate lincRNAs based on CLIP data. Red nodes represent lincRNAs, blue nodes RBPs. Edges are drawn, if significant CLIP sites were found for the respective lincRNA-RBP pair.

So far, our analyses identified general as well as subtype-specific lincRNA expression trends in PDAC that are eventually the net result of gene transcription, RNA processing and decay. These processes are controlled by different classes of proteins, namely transcription factors (TF) and RNA-binding proteins (RBPs). Moreover, small regulatory RNAs, so called microRNAs (miRNAs), are also involved in fine-tuning the expression landscape of cells [48]. In order to gain further insights into the regulatory mechanisms that act in concert to establish specific lincRNA expression patterns, we performed in silico analyses to predict physical interactions. In addition, we retrieved experimental information on RBP-lincRNA binding. First, we focused on putative transcriptional regulators and used the PROMO website (http://alggen.lsi.upc.es/cgi-bin/promo_v3/promo/promoinit.cgi?dirDB=TF_8.3; ref. [49]) to search for human TFs that could bind to the promoter regions of lincRNAs (−2000–+1000 bp up-/downstream of transcription start sites; Figure 4A, Table S5). In total, we found 77 TFs that might control the expression of the 27 selected lincRNAs and in each of those lincRNAs loci we found binding motifs of 35 to 56 different TFs.

Binding motifs of 16 TFs were present in all 27 candidates independent of their expression trend in tumours (up or down) compared to normal tissues. For example, the general transcription factor TBP was predicted to bind to all promoter regions consistent with its role in transcription initiation by all three human RNA polymerases. Other TFs like TP53 and YY1 have been shown to possess dual activity, i.e., these TFs are able to activate and repress transcription and their predicted up- or downregulated lincRNA targets might contribute to the context-dependent tumour-suppressive or oncogenic function of these TFs [50,51]. On the other side, this analysis also revealed some very specific putative interactions. For example, STAT5B motifs were only found in the promoter region of RP3-340N1.2 and it is tempting to speculate about an oncogenic role of this upregulated lincRNA as a downstream target of STAT5B signalling [52].

Furthermore, TEAD2, a TF that plays a key role in the Hippo signalling pathway by interacting with YAP1, was predicted to bind and regulate four lincRNAs (LINC00261, RP11-161M6.2, GMDS-AS1, MIRLET7BHG), three of them showing the strongest decrease in basal-like tumours (Figure 3A). Intriguingly, YAP1 was recently identified as a major driver of the squamous (basal-like) subtype of PDAC and its activation was associated with poor prognosis [53]. However, it remains unclear, if this context-dependent role of YAP1 is, at least partially, dependent on or mediated by lincRNAs and the three candidates identified herein should be further analyzed in the context of Hippo signalling. In addition to these examples, combination of the TF prediction with co-expression analysis (Figure 3B,D) provided additional insights into lincRNA-specific expression control. For example, analysis of the TF binding profiles of AC021218.2 and RP1-193H18.2, whose expression was found to be negatively correlated in the classical subtype, but not in the basal-like, revealed several TFs that might specifically regulate each of the lincRNAs. In detail, AC021218.2 upregulation, particularly found in the classical subtype, might be caused by WT1, USF1, RELA, MYC, ETV4, E2F1, and ARNT whereas downregulation of RP1-193H18.2, more pronounced in the basal-like subtype, might be due to binding of SRY, NFE2, HOXD9, HOXD10, and EBF1 given the mutually exclusive predicted binding sites of these TFs to either one of the lincRNA promoter regions.

In addition to these putative transcriptional regulatory connections, we next considered post-transcriptional mechanisms that might affect lincRNA expression in PDAC. First, we analyzed enhanced crosslinking and immunoprecipitation (eCLIP) data of 153 individual RBPs that were previously generated in K562 and/or HepG2 cells [54,55] and can be downloaded from the ENCODE project website (www.encodeproject.org, ref. [56]). We further included a Photoactivatable-Ribonuleoside-Enhanced Crosslinking and Immunoprecipitation (PAR-CLIP) dataset consisting of two biological replicates containing binding information of the Argonaute protein AGO2 performed in HEK293 cells [57]. In total, we were able to detect physical interactions between eight of the 27 lincRNA candidates and a total of 73 RBPs in these datasets (Figure 4B, Table S6). The overall numbers of lincRNA-RBP interactions ranged from one (IQCH-AS1-QKI, and MAPKAPK5-AS1-DDX3X) to 47 for MIR4435-2HG. The most interactive lincRNAs (MIR4435-2HG, GMDS-AS1, LINC00261) were predicted to associate with more than 20 different RBPs, but only three RBPs had eCLIP peaks in all of them. All three RBPs (PTBP1, HNRNPK, BCLAF1) are known to regulate RNA processing, especially splicing [58,59,60,61]. In accordance with this, gene annotation enrichment analyses (GAEA) using Gene Ontology biological processes and Kyoto Encyclopedia of Genes and Genomes (KEGG) pathway annotations revealed significant enrichments of RBPs associated with splicing for all these three multi-exonic lincRNAs. Furthermore, the eCLIP dataset also included binding information for all three miRNA host genes (MIR4435-2HG, MIRLET7BHG, MIR194-2HG) as well as the MIR4435-2HG paralog LINC00152. As expected, MIRLET7BHG and MIR194-2HG contained significant eCLIP peaks of the ribonuclease DROSHA and its partner DGCR8 that jointly form the nuclear microprocessor complex, which is responsible for cleaving the stem loop of primary miRNA transcripts [62]. Furthermore, these two lincRNAs were associated with AGO2, a core protein of the mammalian RNA-induced silencing complex (RISC). However, the AGO2 CLIP peaks were all found in regions representing mature miRNAs, thus underlining AGO2’s function of binding miRNAs for incorporating them into the RISC [63]. Moreover, eCLIP peaks of LIN28B mapping to MIRLET7BHG was in line with the inhibitory effect of LIN28B on let-7b biogenesis [64]. Interestingly, DGCR8 CLIP peaks were also present in LINC00261 whereas DROSHA CLIP peaks were detectable in LINC00152 suggesting putative interactions between those factors which could be caused by miRNA-like stem loop structures present within these lincRNAs. Indeed, a recent study identified a 121-bp 3′-hairpin structure (M8, see Figure S4A) within LINC00152 and overexpression of this stem loop was sufficient to increase invasion of glioblastoma cells [65]. Sequence alignments revealed that MIR4435-2HG contained the same stem loop sequence. Importantly, analysis of ribosome profiling data indicated that the stem loop interacts with one or more currently unknown RBPs. Thus, we performed a closer examination of the eCLIP data and identified six RBPs that might be able to directly bind to the stem loop sequence or within its close proximity in LINC00152 and MIR4435-2HG, respectively. Out of those six candidate RBPs only eCLIP peaks of YBX3 and PABPC4 consistently mapped to the stem loop region in both lincRNAs (Figure S4B,C). Thus, both RBPs represent compelling candidates for further analysis. For example, YBX3 has been shown to stabilize its mRNA targets [66,67,68] and its putative binding to LINC00152 could contribute to the increased expression of this lincRNA in PDAC, especially in the basal-like subtype.

Next to these protein-based regulatory mechanisms that might control lincRNA expression in pancreatic cancers, we also considered small RNA-mediated regulatory pathways. Hence, we performed miRNA targeting analyses for all 27 lincRNA candidates which yielded a total of 178 miRNAs predicted to bind to six lincRNAs (Figure S5A,B; Table S7). However, when considering only those interactions supported by statistically significant negative correlations in RNA expression (FDR<0.05), only a few putative bindings remained. In the classical subtype, MIRLET7BHG was associated with miR-4736 (ρ=−0.47, FDR=0.01) and MIR4435-2HG was negatively correlated to miR-206 (ρ=−0.38, FDR=0.03). For lincRNA AC021218.2, we observed significant negative correlation with the predicted miRNA binding partners miR-217 (ρ=−0.37, FDR=0.03), miR-202 (ρ=−0.41, FDR=0.01) and miR-20b (ρ=−0.35, FDR=0.04). None of the predicted lincRNA-miRNA interactions showed significant negative correlation in the basal-like subtype samples. While this miRNA target prediction does not exclude the possibility of a regulatory effect of certain miRNAs on our lincRNA candidates in selected cell types or under certain conditions, the limited amount of putative functional miRNA-lincRNA interactions is in line with the RBP interaction analysis which identified robust and reproducible Argonaute eCLIP peaks only for the miRNA host genes MIRLET7BHG and MIR194-2HG. However, eCLIP peaks of TNRC6A, a component of the RNA-induced silencing complex (RISC) and interaction partner of Argonaute proteins, were present in GMDS-AS1 and LINC00152 (see Table S6) hinting towards a loading of both lincRNAs into the RISC and subsequent expression control by selected miRNAs. Interestingly, among all miRNAs predicted to target LINC00152 the strongest negative correlation was observed for miR-206 (see Table S7) which has been shown to regulate the expression of the closely related paralog of LINC00152, namely MIR4435-2HG, in colorectal cancer cells [69]. Moreover, GMDS-AS1 regulation by miR-96-5p might contribute to lung cancer development [70]. Hence, both lincRNAs might be subjected to miRNA-mediated expression control in pancreatic cancer cells too and their regulation by miR-206 or miR-96-5p, respectively, should be analyzed in more detail.

### 2.4. Putative Signalling Pathways and Direct MRNA Targets Associated with LincRNAs

**Figure 5:**
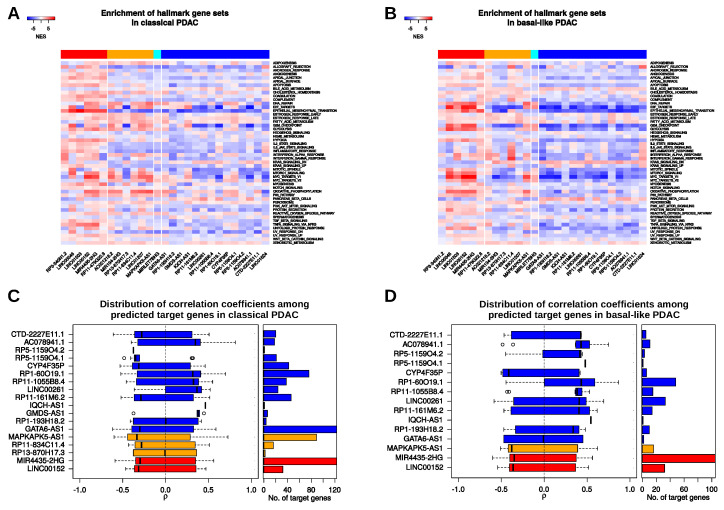
Guilty-by-association studies and potential mRNA-lincRNA-interactions. (**A**,**B**) Normalized enrichment scores (NES) obtained from gene set enrichment analyses (GSEA) using sorted Spearman correlation coefficients (ρ) of protein-coding genes related to the respective 27 lincRNA in classical (**A**) and basal-like (**B**) PDAC samples. Considered were the 50 MSigDB hallmark gene sets. Column color codes denote lincRNAs upregulated in both tumour subtypes compared to normal pancreas with significantly higher expression in the basal-like subtype compared to the classical subtype (red); upregulated in both subtypes and significantly lower in the basal-like subtype (orange); downregulated in both subtypes and significantly higher expressed in the basal-like subtype (cyan); downregulated in both subtypes and significantly lower in the basal-like subtype (blue). (**C**,**D**) Number of predicted mRNA interactors and distribution of the RNA expression correlation coefficients (ρ) of these putative targets and the respective lincRNAs in classical (**C**) and basal-like subtype (**D**) PDAC.

Having identified subtype-specific lincRNA expression patterns as well as putative transcriptional and post-transcriptional regulators or interactors of a selected group of 27 lincRNAs leaves us with the final question of what functions these lincRNAs might have. In order to address this question, we performed a guilty-by-association analysis and calculated the expression correlation between each of the 27 lincRNAs and all protein-coding genes contained in the datasets (18,474) in normal pancreas as well as in the PDAC subtype samples. Subsequently, we used the obtained correlation coefficients to perform GSEA to identify associated signalling pathways (Figure 5A,B) using the MSigDB hallmark gene sets. These 50 gene sets contain genes serving as markers of well-defined biological states or processes [71]. Similar co-expression analyses had been performed in the past to generate hypothesis and to predict diverse roles of lncRNAs, ranging from stem cell pluripotency to cancer [72,73].

Here, our co-expression analysis revealed that all lincRNAs upregulated in PDAC and in addition also significantly upregulated in the basal-like versus the classical subtype showed a positive enrichment of genes associated with epithelial-to-mesenchymal transition (EMT) in both subtypes, whereas a negative enrichment of EMT genes was observed for most of the remaining lincRNAs (Figure 5A,B; Tables S8 and S9). Intriguingly, expression of LINC00152, LINC00346, and LINC01503 was positively correlated with genes that play a role in EMT and all three lincRNAs have been shown to regulate tumour cell migration and invasion, partially via induction of an EMT program in cholangiocarcinoma, gallbladder, gastric, or bladder cancer, respectively [74,75,76,77]. While their relevance for pancreatic cancer is largely unknown, recent studies provided evidence of a function of LINC00346 in pancreatic cancer cell migration and invasion as well as proliferation [78,79].

Hence, it might be worth to investigate the function of other lincRNAs that might positively as well as negatively influence EMT and cell motility. In addition, all upregulated lincRNAs with a higher expression in basal-like PDAC tumours showed a positive enrichment of the apoptosis gene set suggesting additional roles of these lincRNAs in cell death control, which should be further investigated as well. Interestingly, when considering co-expression in classical subtype samples, gene sets related to Interleukin (IL)2 and IL6 signalling showed a rather positive enrichment with the group of lincRNAs that were generally upregulated in PDAC, but higher in basal-like tumour whereas all other lincRNAs returned negative enrichment scores for these two gene sets. Surprisingly, several lincRNAs show a reversed relationship to IL2/IL6 gene sets in the basal-like samples suggesting subtype-specific functions of certain lincRNAs potentially in the context of IL2/IL6 signalling. Several other inversed, i.e., subtype-specific enrichment trends could be found. For example, LINC01207-related correlations showed a significant positive enrichment of genes included in the apoptosis gene set in the classical subtype (NES =1.84, FDR =0.02). However, in the analysis of the basal-like subtype, a negative, yet not significant enrichment (NES =−0.84, FDR =0.68) could be detected lending further support to the idea of subtype-dependent lincRNA functions in PDAC.

Importantly, the correlations and pathway associations observed for these 27 lincRNAs could be both, the cause but also the consequence of several complex molecular interactions. For example, the expression of lincRNAs could be correlated with EMT or apoptosis genes due to overlapping transcriptional or post-transcriptional regulators, e.g., certain TFs, RBPs or miRNAs that jointly regulate the expression of certain lincRNAs and protein-coding genes. On the other side, lincRNAs could sequester or guide TFs as well as RBPs to affect transcription or RNA processing which would result in positive or negative expression correlations. Moreover, lincRNAs could sponge miRNAs to control certain expression programs, again resulting in the observed interdependencies. Importantly, another post-transcriptional mechanism that might be employed by lincRNAs to regulate transcript abundance and therefore might explain positive and negative expression correlations observed herein exists and relies on direct binding of the lincRNA to its respective target mRNA. A prominent example of such a regulatory interaction is found between lncRNA-ATB and IL11 mRNA leading to the stabilization of the latter which results in an activated IL11/STAT3 signalling and enhanced metastatic potential of liver cancer cells [80]. Hence, we wanted to investigate whether such RNA-RNA interactions could also be present among our candidate lincRNAs and their co-regulated protein-coding transcripts in PDAC subtypes. Therefore, we obtained lincRNA-RNA target predictions from a recently developed database [81]. We only considered putative interactions that would result in expression changes of the target transcripts and that were associated with significant (FDR <0.05) Spearman expression correlations. In total, we were able to identify putative mRNA targets of 18 lincRNAs in the classical subtype and of 15 lincRNAs in the basal-like subtype (Figure 5C,D; Tables S10 and S11). In the classical subtype, MIR4435-2HG and GATA6-AS1 both showed significant correlations to 123 predicted target genes, with the majority of these genes having negative correlation coefficients (Figure 5C, Figure S6A). In the basal-like subtype, MIR4435-2HG also had the most predicted bindings (105), again with a trend towards negative expression correlation (Figure 5D). All but two of the predicted interacting mRNAs of GATA6-AS1 showed a significant correlation in the classical subtype, but did not do so in the basal-like subtype (Figure S6B). Also, predicted mRNA interactions of lincRNAs GMDS-AS1, RP11-834C11.4 and RP13-870H17.3 were only significant in the classical subtype. LINC00152, MAPKAPK5-AS1 and CYP4F35P consistently showed trends for negative correlation to predicted targets in both subtypes. Furthermore, AC078941.1, RP1-60O19.1, RP11-1055B8.4 and LINC00261 consistently correlated negatively to their predicted targets. Moreover, individual mRNAs were predicted to interact with two or more lincRNAs suggesting cooperativity or competition among lincRNAs. For example, MUC6 mRNA might be targeted by AC078941.1, CTD-2227E11.1 and RP1-60O19.1 in both subtypes (Figure S6A,B). Of note, all three lincRNAs were downregulated in PDAC, especially in the basal-like subtype and the same expression trend was seen for MUC6 mRNA making it tempting to speculate about a stabilizing interaction between these transcripts. On the other side, SFRP5 mRNA, which encodes a soluble modulator of Wnt signalling and was strongly downregulated in the basal-like subtype might be bound by two lincRNAs with opposite functions. While RP1-60O19.1 showed a strong positive expression correlation, LINC00152 was strongly negatively correlated, suggesting stabilizing or destabilizing effects of their interaction with SFRP5 mRNA, respectively. In general, we could observe that lincRNAs upregulated in the PDAC samples tended to be negatively correlated to their predicted target transcripts in both subtypes suggesting rather destabilizing functions among our candidate lincRNA which should be further investigated in the future.

Last but not least, we performed a preliminary analysis to investigate the impact of genetic activation of oncogenic signalling pathways on lincRNA expression. To this end, we focused on KRAS and compared the differential expression of lincRNAs between TCGA PDAC samples with wildtype KRAS (wt, *n* = 10) against those samples with activated, mutant KRAS (mut, *n* = 139). Applying the same criteria for the determination of differential expression (i.e., FDR <0.05; $|log_{2}FC|>1$; minimum average CPM >5 in either KRAS wt or mut samples), we identified only seven lincRNAs differentially expressed between these two conditions (Table S12). Interestingly, three of these were also found in our list of 27 lincRNAs significantly deregulated between the PDAC subtypes, namely RP1-193H18.2 ($log_{2}FC$ mut/wt =−1.37), RP3-340N1.2 ($log_{2}FC$ mut/wt = 3.76), and LINC00261 ($log_{2}FC$ mut/wt =−3.56). Interestingly, co-expressed genes of these three lincRNAs showed significant positive enrichment of the KRAS_SIGNALING_DN gene set as well as a significant negative enrichment of the KRAS_SIGNALING_UP gene set, which was also dependent on the PDAC subtype (see Figure 5A,B). Of note, although the number of KRAS wt samples was very low, most of them (8/10) had been assigned to the classical subtype, whereas only two of them were classified as basal-like, which might partially explain the subtype-dependent enrichment of co-expressed KRAS target genes. Importantly, this preliminary analysis revealed that certain lincRNA expression patterns might be caused by individual tumour-associated mutations, which warrants further investigations.

## 3. Discussion

Long non-coding RNAs are a large and heterogeneous group of transcripts that play a role in diverse biological systems. Although their general contributions to multiple human diseases, e.g., cancer, have been described in recent years, many questions remain. For example, gene expression analyses performed on thousands of tumour samples from a broad range of tumour entities have identified cancer subtypes that are defined by certain gene expression pattern [82,83,84,85]. Also lncRNAs have been assigned to these molecular subtypes, however, it is unclear, if they play an active role in shaping the expression landscape of these subtype and/or whether these lncRNAs have subtype-specific functions as well as interaction partners. Here, we provide a comprehensive analysis of lincRNAs in pancreatic cancer and we highlighted several interesting findings and generated hypotheses about the regulation and function of selected lincRNAs that require further validations as well as experimental interrogation. For example, our differential expression analysis revealed a strong reduction of NEAT1 and MALAT1, two highly conserved nuclear lncRNAs [34,35]. Interestingly, the role of NEAT1 in pancreatic cancer seems to be controversial. Loss of Neat1 in a KrasG12D-driven mouse model of pancreatic cancer has been shown to promote the development of premalignant pancreatic intraepithelial neoplasia (PanIN) and cystic lesions [86]. On the contrary, several studies claim that NEAT1 acts through direct interaction with specific mRNAs (e.g., ELF3) or via sponging certain miRNAs thereby promoting cancer cell growth, invasion, and migration [87,88,89,90]. Based on the expression changes observed in our analysis, it is tempting to speculate that NEAT1 might indeed function as a tumour suppressor in PDAC. In addition, studies on MALAT1 in pancreatic cancer largely suggest an oncogenic function of this lincRNA. For example, MALAT1 was found to be upregulated in tumours compared to adjacent normal tissue and its downregulation inhibited cell proliferation, migration, invasion, and promoted apoptosis in pancreatic cancer cell lines [91]. Yet, no effect on tumour progression upon Malat1 depletion was observed in a highly aggressive mouse model of pancreatic cancer [92]. Importantly, the general role of MALAT1 acting as a common oncogene and driver of metastasis across multiple cancer types is currently debated and genetic studies in breast cancer indicated that it could also act as a tumour suppressor [93,94,95,96]. Our finding of a significantly reduced expression of MALAT1 in PDAC is partially contradictory to a previous study that analyzed the clinical significance of MALAT1 in pancreatic cancer using multiple datasets from different public databases (GEO, Oncomine, TCGA) [97]. However, the same study also found heterogeneity among the data with at least three other datasets (GSE3654, GSE16515, GSE1542) showing no significant expression differences [98,99,100]. Therefore, more sophisticated genetic tools and targeting strategies as well as more advanced cell-based model systems (e.g., patient-derived xenografts, organoids, refs. [101,102]) should be used in the future to dissect the molecular functions of MALAT1 and NEAT1 in pancreatic cancer.

In addition, our study found multiple other lincRNAs to be deregulated in PDAC subtypes and with currently unknown functions. However, a limitation of our analysis pipeline is the use of RNA-seq data from different sources (normal pancreas from the GTEx and PDAC from the TCGA project) to identify putative candidate lincRNAs for further investigations. Although we used raw count data from these two sources and normalized the samples together to avoid RNA composition biases, we cannot exclude batch effects that might arise from, e.g., differences in sample handling and processing. Wang et al. [103] proposed a pipeline for uniform processing and batch effect removal for comparing GTEx and TCGA data. The validation of this empirical correction method, however, relies on comparing adjusted expression values of normal tissue samples provided by the TCGA and the GTEx samples. Since the TCGA PDAC project offers only four normal pancreas samples, Wang et al. considered the TCGA PDAC cohort to be not suited to be processed with their pipeline. Furthermore, this method requires the TCGA normal tissues to represent adequate controls. However, these samples were collected from sites neighbouring cancerous tissue and thus might also be affected by deregulated gene expression. Thus, we did not adjust the data to account for batch effects. Nevertheless, our results are in line with previously reported findings. For example, GSEA performed on differential gene expression results confirmed that numerous protein coding genes known to be deregulated in pancreatic cancer were determined as such (Figure S1A). Further, we could confirm several reports about deregulation of specific lincRNAs. For example, we found MEG3 to be downregulated as reported in previous pancreatic cancer studies [28,29,30,31]. Upregulation of LINC01207 and LINC00152/CYTOR in PDAC was also reported previously [39,45]. Recently, we validated the herein proposed downregulation of LINC00261 by comparing RNA expression of 34 normal pancreatic tissue samples with 42 PDAC samples [43]. Further functional analyses revealed a connection of this lincRNA to the EMT programme, which we also propose herein based on the guilty-by association studies performed with GSEA. Altogether, we believe that our analyses provided a reasonable starting point for the identification of lincRNAs of particular interest for further PDAC research. However, the validation of the deregulation of each candidate lincRNA remains imperative.

Another area that requires much more attention in the future is the identification of genetic, epigenetic and post-transcriptional mechanisms that control lincRNA expression and regulation in PDAC subtypes. While we presented a broad analysis of putative connections between lincRNAs and TFs, as well as RBPs and miRNAs, which need to be validated experimentally, we did not extensively consider genetic and epigenetic factors that could explain some of the observed expression differences. Yet, our preliminary analysis of the impact of KRAS mutation status revealed that certain lincRNA expression patterns might be caused by individual tumour-associated mutations. However, additional chromosomal alterations (amplifications, deletions, rearrangements) might as well contribute to the differential expression of individual lincRNAs. Another factor that could cause general or subtype-specific expression differences is DNA methylation, which could lead to epigenetic gene silencing. In fact, GATA6-AS1 (and GATA6) expression was shown to be controlled by two distinct mechanisms: lower expression in basal-like tumours might be caused by higher DNA methylation near the GATA6 gene, whereas classical tumours showed copy number gains of the GATA6 neighbourhood in conjunction with higher expression of GATA6 mRNA as well as GATA6-AS1 in the classical subtype [3]. Intriguingly, there seems to be a positive or negative, subtype-specific selection pressure on the GATA6/GATA6-AS1 gene locus confirming an important and complex role for both genes in PDAC and other cancers [104,105,106].

In summary, our study provides a comprehensive list of subtype-associated lincRNAs that might directly or indirectly contribute to subtype-specific transcription programs and cellular phenotypes. To understand the regulatory roles of these lincRNAs in more detail, comprehensive gain or loss of function experiments in a broad selection of in vitro and in vivo model systems need to be performed in the future.

## 4. Materials And Methods

### 4.1. RNA-Seq Data Processing, Differential Expression and Survival Analysis

We obtained gene-level RNA-seq read counts of TCGA primary tumour PDAC samples and GTEx V7 normal pancreas tissue via the GDC data portal (portal.gdc.cancer.gov) and the GTEx portal (gtexportal.org), respectively. By combining these data, we got read count information of 53,045 genes for 177 primary tumour samples and 248 normal pancreas tissue samples. Differential gene expression was assessed using R/edgeR v3.28.0 after normalizing all samples together applying the trimmed mean of M-values (TMM) normalization method [107,108]. CPM transformation was utilized to obtain normalized expression values. The classification of the TCGA tumour samples into PDAC subtypes was taken from [3]. LincRNA information was gained by extracting genes termed as lincRNAs according to ENSEMBL v38.86 annotation [109]. MiRNA expression data were also obtained by downloading read count data of TCGA PDAC samples via the GDC data portal. CPM values were generated after applying TMM normalization using edgeR. Normalized expression data (RPKM) from cell lines were obtained from the CCLE (https://portals.broadinstitute.org/ccle/data; version 20180929; ref. [37]). Classification of PDAC cell lines into basal-like or classical subtype origin were obtained from [38]. Only cell lines associated to a subtype class with a false discovery rate (FDR) value less than 0.05 were considered.

### 4.2. LincRNA Localization Predictions

Subcellular localizations were predicted using DeepLncRNA [110]. For each lincRNA localizations of all ENSEMBL v38.86 transcripts were predicted. Terms “Cytosol” and “Nuclear” indicate that all transcript isoforms of a particular lincRNA gene were predicted to be localized in the cytosol or nucleus, respectively. Term “both” indicates that transcript variants of the same lincRNA gene were predicted to be localized in either the cytosol or the nucleus.

### 4.3. Enrichment Analyses

Gene set enrichment analyses (GSEA) were performed using the GSEA v3.0 software [24] and MSigDB v7.0 gene sets [71], applying the pre-ranked test, 1000 permutations and the classical scoring scheme.

### 4.4. RBP Binding Predictions

ECLIP data of 153 RNA binding proteins were obtained from the ENCODE project website (www.encodeproject.org [56]). These eCLIP experiments were performed in Hep-G2 or K-562 cells and always comprised two biological replicates. Statistically significant peaks (score = 1000) were extracted from the downloaded narrow peak bed files (GRCh38) and intersections of these peaks with lincRNAs were determined using bedtools v.2.25.0 [111]. Only if significant peaks for a particular lincRNA were found in both replicates of an experiment, an RBP was considered a putative binding partner of the respective lincRNA.

Peak files (hg19) of two replicates of an AGO2 PAR-CLIP experiment (GEO accession GSM714644 and GSM714645 [57]) were obtained from the CLIP-DB website (lulab.life.tsinghua.edu.cn/clipdb [112]). Putative binding to lincRNAs was determined as described for the eCLIP data.

### 4.5. MiRNA Binding Prediction

To infer putative lincRNA-miRNA bindings, we first queried for predicted miRNA bindings utilizing the R-package multiMiR v1.8.0 [113] using all eight prediction databases and the default prediction cutoff of 20%. We chose a particular lincRNA-miRNA interaction, if it was reported by at least one of the databases.

### 4.6. LincRNA-MRNA Target Predictions

LincRNA target predictions were obtained from the database accessible at http://rtools.cbrc.jp/cgi-bin/RNARNA/ [81]. For each transcript isoform of a certain lincRNA, we gathered the top 100 prediction results ranked by minimum energy and extracted the unique gene identifiers of the predictions of all isoforms as putative targets.

### 4.7. TF Binding Prediction

Putative transcription factor binding sites were determined using the PROMO Website (alggen.lsi.upc.es/cgi-bin/promov3/promo [49]) searching for human transcription factors at a maximum matrix dissimilarity of 5%. Input genomic sequences starting 2000 bp upstream of the transcription start and ranging 1000 bp into the gene body were obtained from ENSEMBL v38.86 via the biomaRt v2.42.0 R/Bioconductor-package [114,115].

### 4.8. Survival Analysis

Prognostic relevance of lincRNA expression to predict overall and disease-free survival (OS and DFS, respectively) of PDAC patients was assessed using the GEPIA2 data portal (gepia2.cancer-pku.cn [36]). Basal and/or classical subtype samples from the TCGA PAAD dataset were selected to run the analysis for each lincRNA separately. Log-rank test (Mantel-Cox) was applied by GEPIA2 for hypothesis testing. The cox proportional hazard ratio (HR), logrank *p*-value and the 95% confidence interval information was included in the respective Kaplan-Meier graphs.

## 5. Conclusions

We systematically investigated the expression of lincRNAs in pancreatic cancer and its molecular subtypes using publicly available data from large-scale studies. We identified several deregulated lincRNAs which also showed significant different expression patterns across PDAC subtypes. Subsequent expression correlation analyses implied that subtype-associated lincRNAs might directly or indirectly contribute to subtype-specific transcription programs and regulate cellular processes like migration or apoptosis. Mechanistically, we propose that lincRNAs might be regulated by transcriptional as well as post-transcriptional regulators. We predicted putative TFs as well as interacting RBPs, miRNAs and mRNAs that might be targeted by lincRNAs or might affect their expression. In summary, we identified several PDAC-associated lincRNAs of prognostic relevance and potential context-dependent functions and molecular interactions. Hence, our study provides a valuable resource for future investigations to decipher the role of lincRNAs in pancreatic cancer.

## Supplementary Materials

The following are available online at https://www.mdpi.com/2072-6694/12/8/2077/s1, Figure S1: Differential expression of lincRNAs in PDAC subtypes, Figure S2: LincRNAs with prognostic relevance across all TCGA PAAD samples, Figure S3: LincRNAs with subtype-specific prognostic relevance in TCGA PAAD samples, Figure S4: M8 stem loop and its putative interaction partners, Figure S5: Potential targeting of selected lincRNAs by microRNAs, Figure S6: Putative interactions between selected lincRNAs and protein-coding transcripts, Table S1: Differential expression of lincRNAs in PDAC, Table S2: LincRNA expression in PDAC-derived cell lines, Table S3: 27 selected lincRNA candidates, Table S4: Correlation coefficients of the 27 selected lincRNA candidates, Table S5: Predicted TF bindings, Table S6: Predicted RNA RBP interactions, Table S7: Predicted miRNA interactions, Table S8: GSEA results of correlation between selected lincRNAs and protein coding genes in classical PDAC, Table S9: GSEA results of correlation between selected lincRNAs and protein coding genes in basal-like PDAC, Table S10: Predicted mRNA interactions in classical PDAC, Table S11: Predicted mRNA interactions in basal-like PDAC, Table S12: Differential expression of lincRNAs in KRAS wild type vs KRAS mut PDAC samples.
