# Supplementary material for: Comprehensive Analysis of LincRNAs in Classical and Basal-Like Subtypes of Pancreatic Cancer
Source: Cancers (Basel). 2020 Jul 27;12(8):2077. doi: 10.3390/cancers12082077 (PMC7464731; doi:10.3390/cancers12082077)
Supplement: Supplementary file 1 [file cancers-12-02077-s001.zip › Supplementary Material/Supplementary Figures.pdf]

cancers

MDPI

Article

Comprehensive Analysis of LincRNAs in Classical and Basal-Like Subtypes of Pancreatic Cancer

Markus Glaß, Agnes Dorn, Stefan Hüttelmaier, Monika Haemmerle and Tony Gutschner

Supplementary Materials

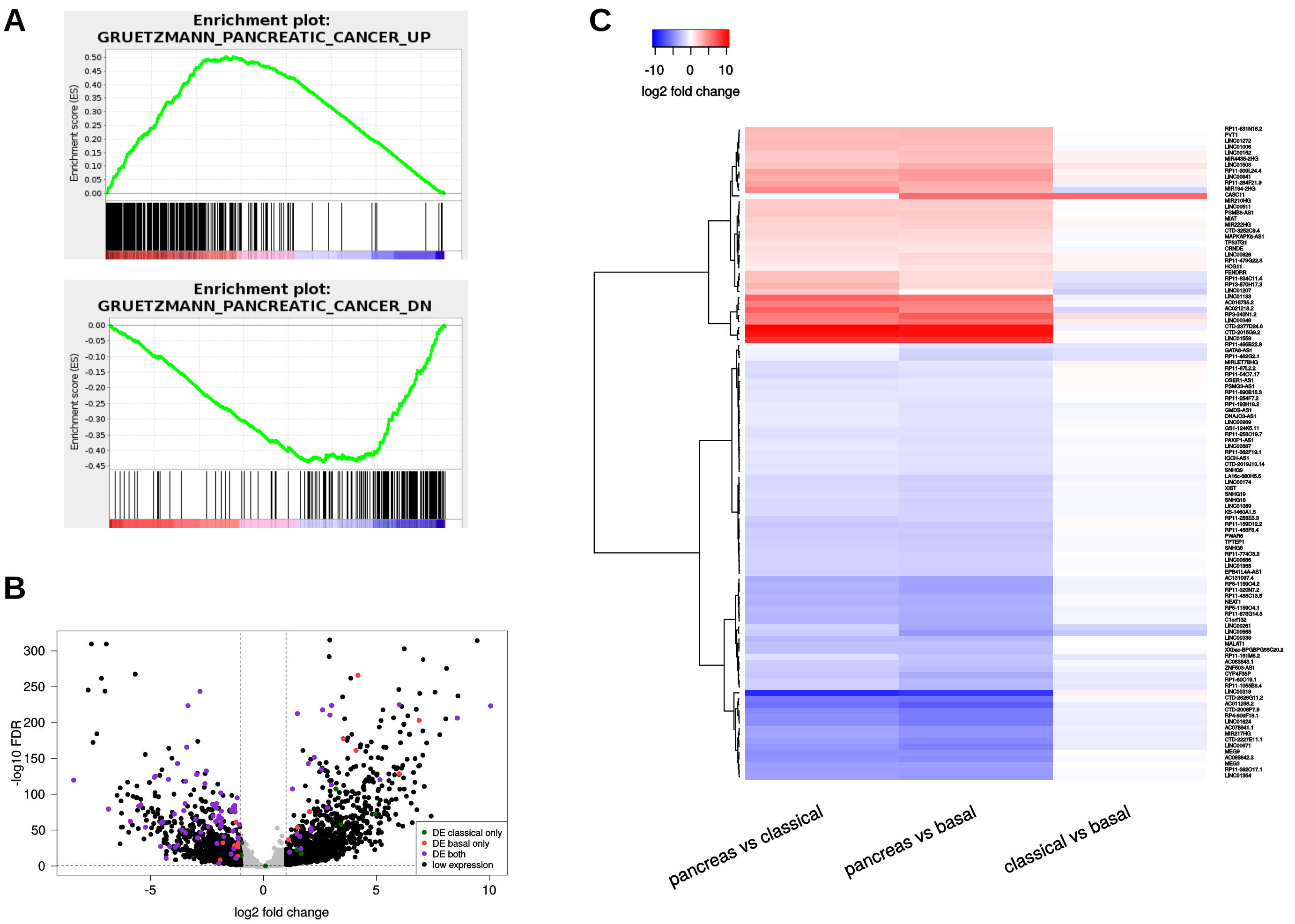

Figure S1. Differential expression of lincRNAs in PDAC subtypes. (**A**) Gene set enrichment analysis (GSEA) applied to expression changes of protein-coding genes found in the comparison of normal pancreatic tissue vs basal-like PDAC subtype samples. Top: Genes known to be upregulated in pancreatic cancer tended to be upregulated in the comparison GTEx pancreas vs TCGA basal-like PDAC. Normalized enrichment score (NES) = 10:76; FDR q-value = 0.0. Bottom: Genes known to be downregulated in pancreatic cancer tended to be downregulated in the comparison GTEx pancreas vs TCGA basal-like PDAC. NES = −7:02; FDR q-value = 0.0. (**B**) Volcano plot showing the distribution of the fold changes and false discovery rates (FDR) of all annotated lincRNAs (*n* = 6899) in the comparison between normal pancreas and basal-like PDAC samples. Purple points mark lincRNAs determined differentially expressed in the comparison between pancreas and basal-like as well as between pancreas and classical PDAC samples. Orange points mark lincRNAs exclusively deregulated in the basal-like subtype. Green points mark lincRNAs differentially expressed in the classical subtype only. Black points denote lincRNAs whose average expression values were below 5 CPM in normal pancreas and in the respective PDAC subtype samples and thus were not considered as differentially expressed although their *log2* fold changes and FDR values met the required criteria (*/log2FC/* > 1; *FDR* < 0.05). (C) Heatmap showing *log2* fold changes of all 109 lincRNAs differentially expressed in classical and/or basal-like PDAC samples compared to normal pancreas tissue.

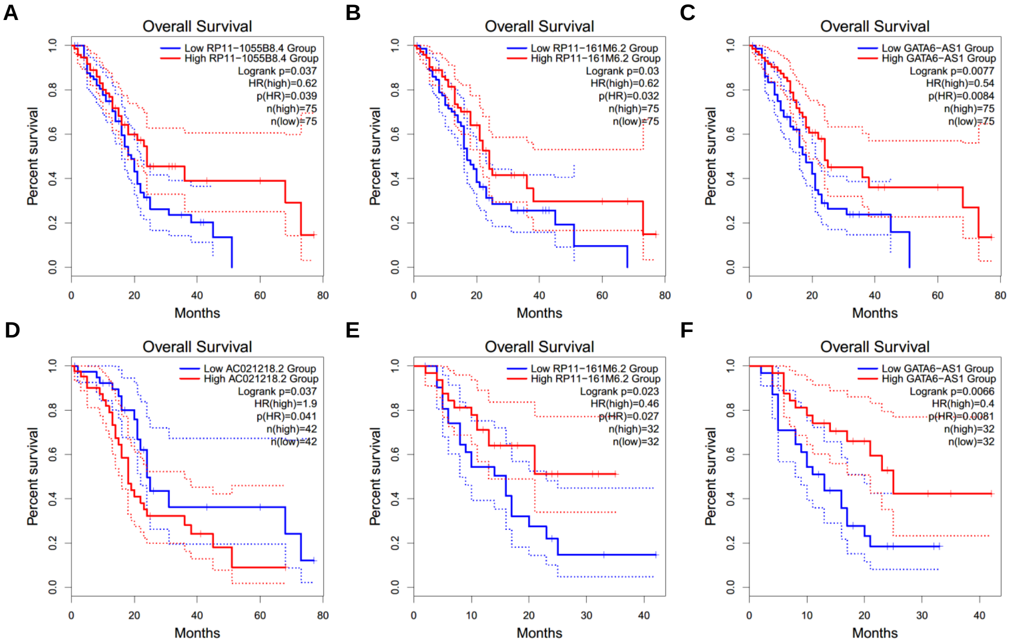

Figure S2. LincRNAs with prognostic relevance for overall survival of PAAD patients. (**A**–**F**) Overall survival analysis (OS) based on lincRNA expression was performed on both subtypes together (n = 150; TCGA PAAD samples) or each subtype individually (n = 84 samples for classical subtype; n = 64 samples for basal subtype) using GEPIA2 (gepia2.cancer-pku.cn). Log-rank test (Mantel-Cox) was used for hypothesis testing. The cox proportional hazard ratio (HR), logrank p-value and the 95% confidence interval information is shown. Low expression of (**A**) RP11-1055B8.4, (**B**) RP11-161M6.2, and (**C**) GATA6-AS1 is generally associated with worse OS across TCGA PAAD samples. In contrast, high expression of (**D**) AC021218.2 is associated with poor OS of PDAC patients assigned to the classical subtype, whereas low expression of (**E**) RP11-161M6.2 and (**F**) GATA6-AS1 specifically predicts poor OS in the basal subtype.

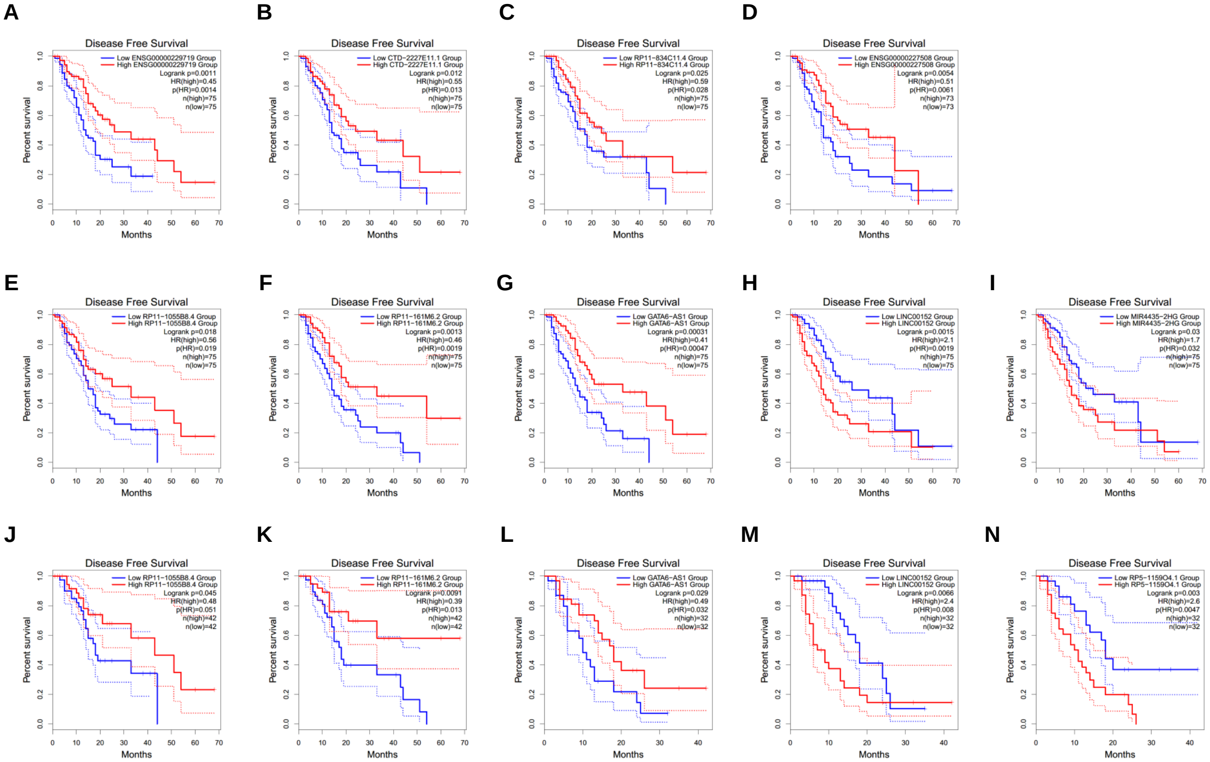

Figure S3: LincRNAs with prognostic relevance for disease-free survival of PAAD patients. (**A**–**N**) Disease-free survival analysis (DFS) based on lincRNA expression was performed on both subtypes together (**A**–**I**; 150 TCGA PAAD samples) or each subtype individually (**J**,**K**, classical subtype, *n* = 84 samples; **L**–**N**, basal subtype, *n* = 64 samples) using GEPIA2 (gepia2.cancer-pku.cn). Low expression of ENSG00000229719/MIR194-2HG (**A**), CTD-2227E11.1 (**B**), RP11-834C11.4 (**C**), ENSG00000227508/LINC01624 (**D**), RP11-1055B8.4 (**E**), RP11-161M6.2 (**F**), and GATA6-AS1 (**G**) as well as high expression of LINC00152 (**H**) and MIR4435-2HG (**I**) is associated with poor DFS across PDAC patients. In the classical subtype, low levels of RP11-1055B8.4 (**J**) and RP11-161M6.2 (**K**) are predictive of poor DFS, whereas in the basal subtype low expression of GATA6-AS1 (**L**) as well as high expression of LINC00152 (**M**) and RP5-1159O4.1 (**N**) are associated with worse DFS.

A

5'…CCTCTACCTGTTGCCCGCCGATCACAGCCGGAATGCAGCTGAAAGATTCCCTGGGGCCTG
GTTCCAACCGCCCACTGTGGACTCTGAGGCCTCTGCATTTGCGGGTGGTCTGCCTGTGATA…3'

B

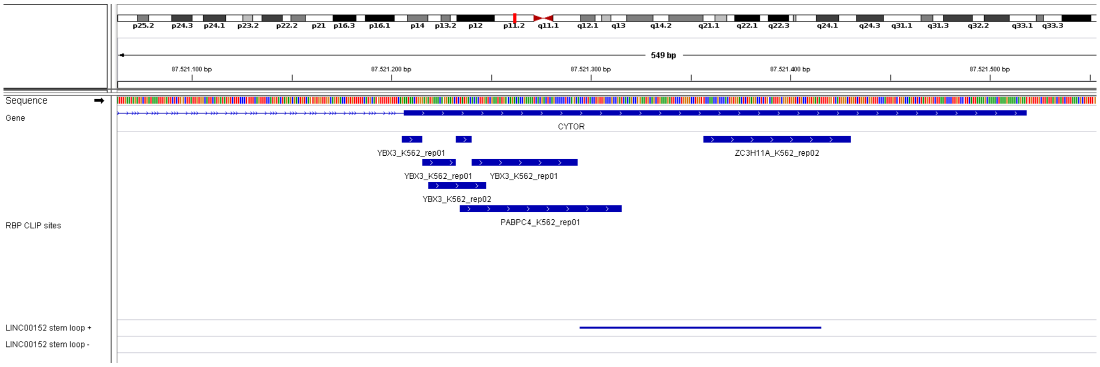

C

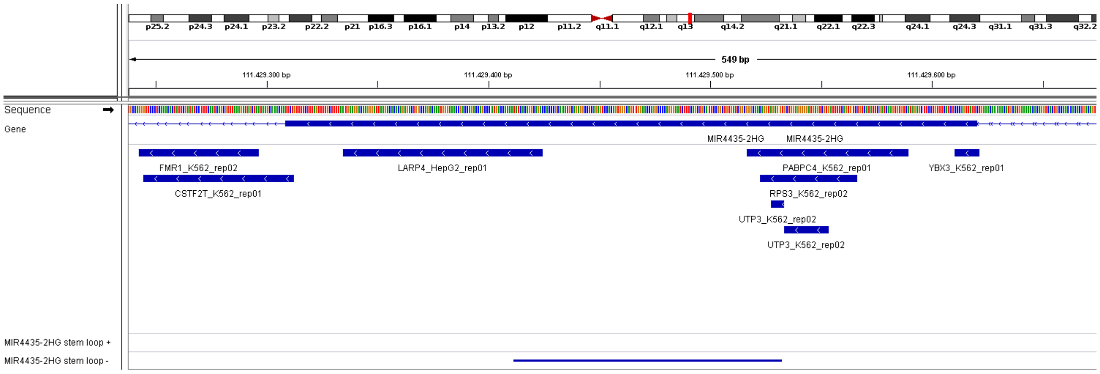

Figure S4: M8 stem loop and its putative interaction partners. (**A**) Sequence of M8 stem loop present in LINC00152. (**B**) Distribution of eCLIP peaks of RBPs across the stem loop region (blue line) in LINC00152 (CYTOR). (**C**) eCLIP peaks of RBPs across the stem loop region (blue line) in MIR4435-2HG.

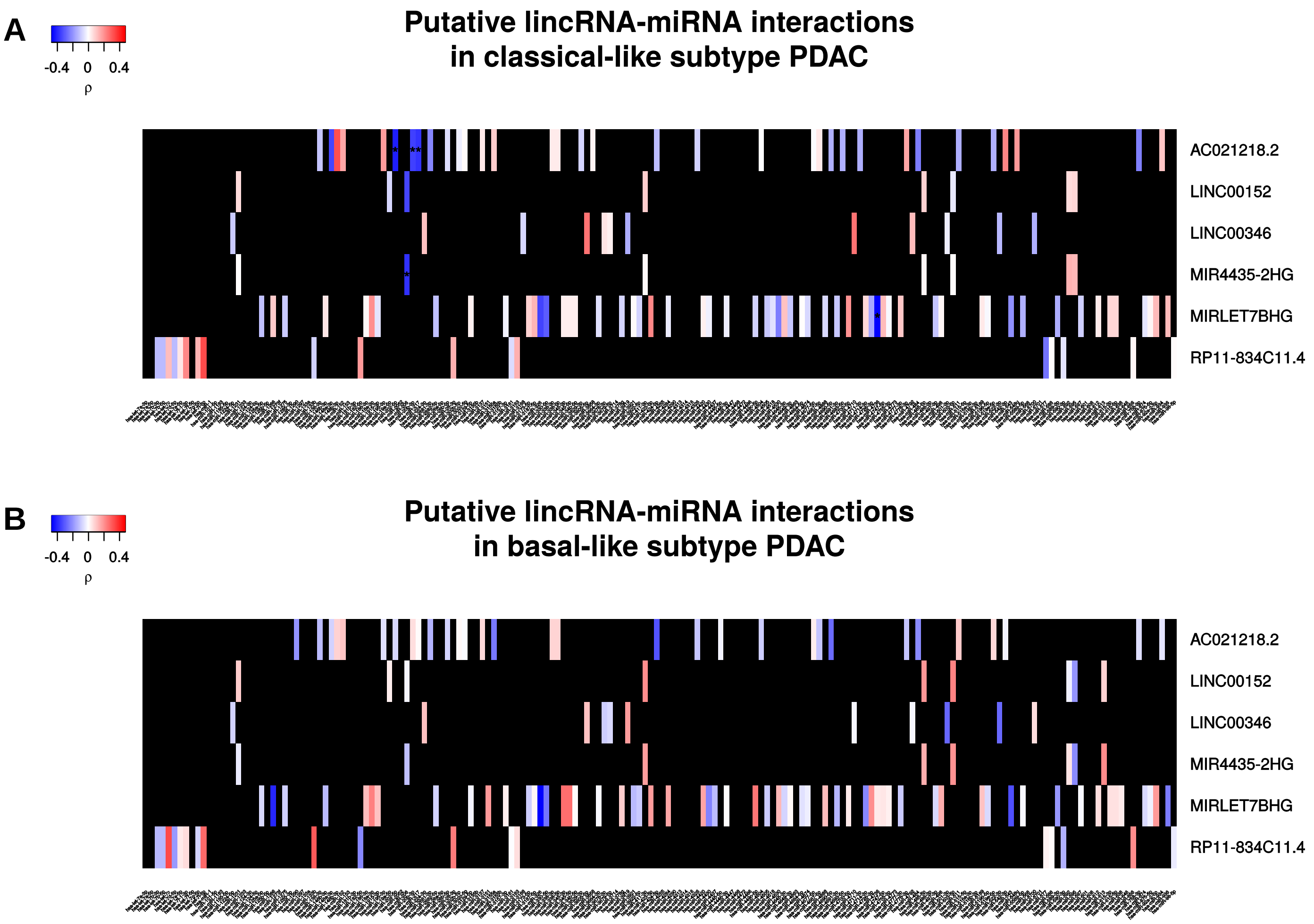

Figure S5: Potential targeting of selected lincRNAs by microRNAs. Predicted lincRNA-microRNA inter-actions and associated Spearman correlation coefficients (ρ) in classical (**A**) or basal-like (**B**) PDAC subtype samples. "*" indicates significant ($FDR < 0.05$) Spearman correlation.

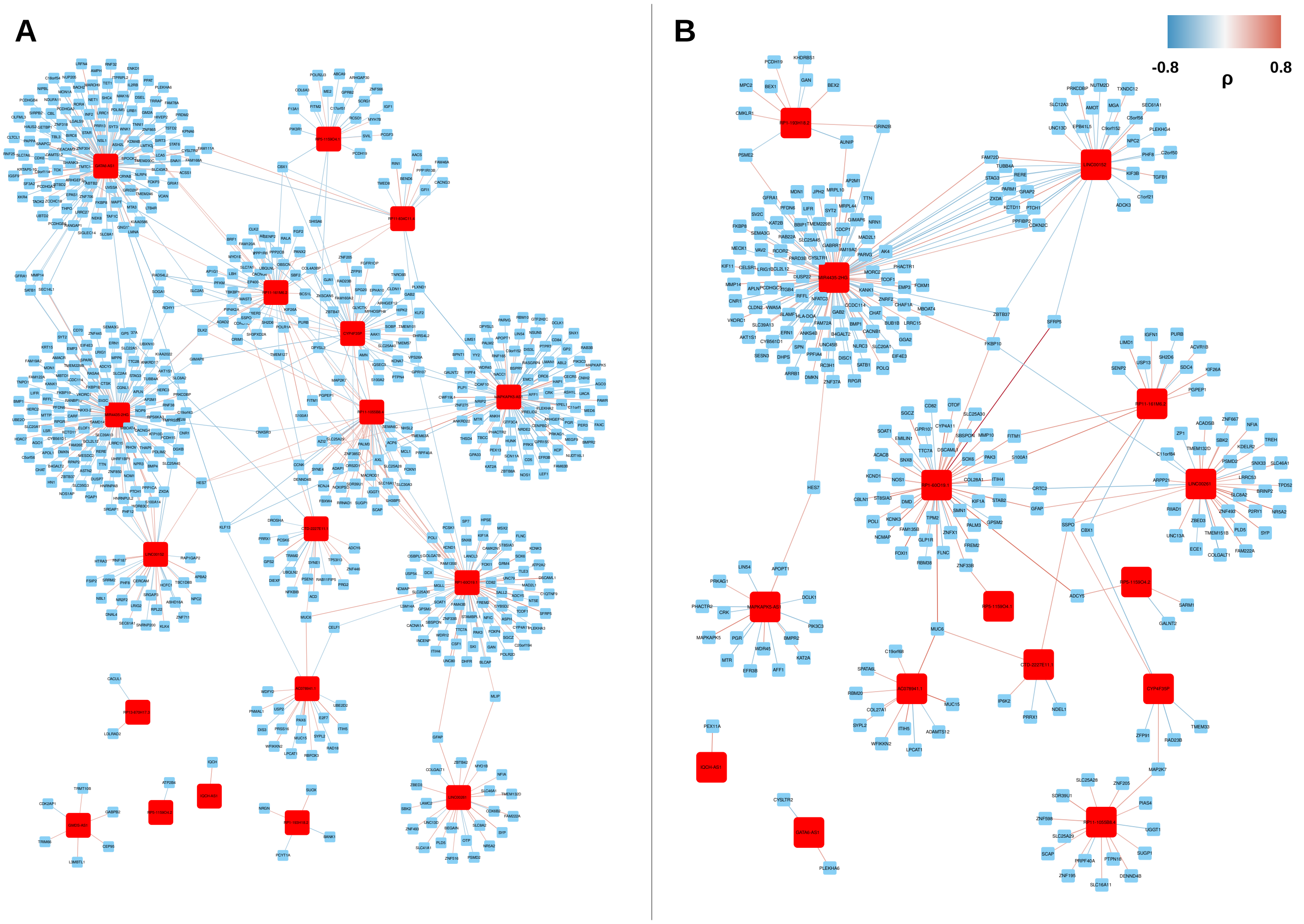

Figure S6: Putative interactions between selected lincRNAs and protein-coding transcripts. Red nodes represent lincRNAs and blue nodes mRNAs predicted to be targets of the lincRNAs. Edges are drawn if an interaction between the respective lincRNA-mRNA pair was predicted and associated with a significant (*FDR* < 0.05) positive or negative correlation in RNA expression in classical (**A**) or basal-like (**B**) PDAC subtypes. Edge colors reflect the correlation coefficients.
